# Comparing the effects of intraocular pressure and tear production measurements in horses in two different environments: Horse stable and medical barn

**DOI:** 10.1111/evj.14067

**Published:** 2024-01-24

**Authors:** Harun Cinar, Latif Emrah Yanmaz, Nurefsan Buyukkaraca, Zehranur Kaya, Mirkan Kosuncu

**Affiliations:** ^1^ Department of Surgery, Faculty of Veterinary Medicine Burdur Mehmet Akif Ersoy University Burdur Turkey

**Keywords:** environment, eye, horse, Schirmer, stress

## Abstract

**Background:**

To date, there are no studies on the impact of two distinct environments—one familiar to the horse and another unfamiliar—on intraocular pressure (IOP) and tear production.

**Objectives:**

To compare the measured IOP and tear production values in horses between a horse stable and a medical barn.

**Study design:**

Cross‐over.

**Methods:**

Thirty healthy male Arabian horses, aged 6.88 ± 3.34 years were used. IOP and tear production measurements were assessed in both the horse stable and the medical barn, with a paired Student's *t*‐test and Bland–Altman analysis conducted for comparison and agreement, respectively.

**Results:**

A significant increase in IOP was observed in the medical barn (34.2 ± 6.8 mmHg) compared with the horse stable (29.5 ± 7.2 mmHg, *p* = 0.02). However, no statistically significant difference in tear production was found between horse stable (22.1 ± 2.8 mm/min) and medical barn (23.6 ± 3.4 mm/min) (*p* = 0.09). The standard error of the slope was 0.36 for the IOP measured in the medical barn, indicating a difference of −4.7 mmHg compared with the IOP measured in the horse stable (*p* = 0.02). The bias was fitted to *y* = −7.9350 + 0.1003*x*. The standard error of the slope was 0.39 for the tear production measured in the medical barn, indicating a difference of −1.5 mm/min compared with the tear production measured in the horse stable (*p* = 0.09). The bias was fitted to *y* = 6.1530 + −0.3367*x*.

**Main limitations:**

The absence of horses with ocular disorders and an assessment of the potential impact of transportation.

**Conclusions:**

A notable increase in IOP was observed in the medical barn compared with the horse stable, while tear production exhibited no significant variance between the two environments. The Bland–Altman analysis highlighted a discrepancy in IOP measurements in the horse stable, emphasising the potential influence of the environment on ocular parameters in horses.

## 1 INTRODUCTION

In the evaluation and maintenance of ocular health, both intraocular pressure (IOP) and tear production play crucial roles. Elevated IOP is often a key indicator of glaucoma, which is associated with optic nerve damage and vision impairment. Conversely, reduced IOP may signal conditions such as hypotony, with potential implications for visual health. Furthermore, an imbalance in tear production is linked to dry eye syndrome, an ocular disorder characterised by discomfort, irritation, and the potential for corneal damage.^1^

Horses are sensitive to stress, and elevated stress levels may induce changes in their behavioural and physiological parameters. These alterations can manifest in variations in the form and frequency of behavioural responses, and fluctuations in heart rate, body temperature, hormones, and other blood indicators, all of which are measurable.^2^ Since horse stables function as the principal living arrangement^3^ for diverse categories of horses accustomed to stalled environments—such as those employed for riding, competition, or labour—it is plausible that these animals experience reduced stress levels within these settings. In contrast, environments where medical procedures are conducted, such as medical barns or hospitals, can be expected to involve various stress factors.

Previous studies in dogs,^4^ rabbits,^5^ and humans^6^ have reported significantly higher IOP measurements in the first eye compared with the second eye. These consistent findings in different species may be related to stress or anxiety induced by an unfamiliar environment or procedure.^4^ Additionally, tear production can decrease due to autonomic control during stress.^7^ However, no equine study has explored the effects of two different environments—one to which the horse is accustomed and another to which it is not accustomed—on IOP and tear production recordings. Therefore, this study aimed to compare IOP and tear production values measured in horses that were measured in a horse stable and a medical barn. Our hypothesis was that there are significant differences in IOP and tear production measurements between horse stable and medical barn, potentially influencing clinical ophthalmologic examinations in horses.

## 2 MATERIALS AND METHODS

The study occurred in the Mediterranean region of Turkey, specifically in the city of Isparta. It involved 30 healthy male Arabian horses, aged 6.88 ± 3.34 years and took place over 2 days in November 2023. The medical barn had recently been constructed, and the horses had not been in the medical barn before the study. Both the horse stable and medical barn were enclosed spaces with identical conditions of light, temperature, and relative humidity, positioned 50 m apart. Horses underwent a 3‐day acclimation period to periocular handling before the commencement of the experiment.

Prior to initiating the study, a comprehensive evaluation was conducted on all horses, comprising a detailed physical examination, complete ophthalmic assessment, and a basic blood profile. The ophthalmic examination encompassed various evaluations, including the menace response, dazzle reflex, pupillary light reflex, direct ophthalmoscopy (Heine mini 3000 F.O, Heine Optotechnik), rebound tonometry (Icare, Tonovet Plus), Schirmer tear test‐1 (Tear Touch, Madhu Instrument Pvt. Ltd.), and fluorescein staining. Only horses determined to be in good health and free from any pre‐existing ocular or systemic diseases were included in this study.

The farm was visited twice, with a 1‐day interval between visits, to collect IOP and tear production readings. On the initial farm visit, IOP and tear production measurements were initially assessed for 15 horses in the horse stable, while another 15 horses were first evaluated in the medical barn. On the second day of the visit, the groups were swapped, meaning that horses initially evaluated in stalls during the first visit were now assessed in the medical barn, and vice versa for the other group. Horses were acclimated to each environment 15 min prior to the commencement of measurements. A 2‐h interval was maintained for the same horse between measuring IOP and tear production in the horse stable and the medical barn. The horses accessed both environments by walking alongside the horse groom.

Horses were maintained in a standing position with their heads positioned above the withers.^8^ All IOP measurements were conducted by the same examiner, proficient in using the Tonovet Plus (Icare Finland Oy), following the manufacturer's guidelines. The tonometer was set to the horse setting, and readings were obtained between 11:00 AM and 3:00 PM. No sedatives, local anaesthetic blocks, or topical ocular anaesthesia were administered. The upper eyelid was gently held without exerting digital pressure on the eye. After adjusting the tonometer, six repeated IOP readings were obtained by gently pressing the tonometer button. Readings displaying ‘yellow’ or ‘red’ were excluded, and only those presented in green were included in the analysis. The sequence of IOP measurements for the left and right eyes was determined randomly through a coin flip. After recording the IOP for each horse, tear production measurements were undertaken after a 5‐min interval. The Schirmer tear test strip, without the use of local anaesthetics, was inserted into the lateral third of the inferior conjunctival fornix for 1 min in both eyes to measure TP. The examiner manually maintained eyelid closure during tear production measurements.

### 2.1 Data analysis

The required sample size was determined using G*Power 3.1,^9^ with a significance level set at 5% and a power of 90%. Statistical analysis was conducted using MedCalc statistical software. A visual assessment of a Q‐Q plot, coupled with the Shapiro–Wilk test, was employed to validate the normal distribution. Tests assessing normal distribution were conducted to ascertain the degree of skewness. To examine statistical differences between the IOP and tear production values of the left and right eyes, we employed the paired Student's *t*‐test. Following this, to compare IOP and tear production values between horse stable and medical barn, an average of the IOP and tear production values for both eyes was calculated, and a paired Student's *t*‐test was performed for comparison. The agreement between IOP and tear production, measured in the medical barn, and IOP and tear production measured in the horse stable were assessed by the use of the Bland–Altman method.^10^ A significance level of *p* < 0.05 was considered statistically significant. Data were presented as mean ± standard deviation.

## 3 RESULTS

All horses completed IOP and tear production measurements successfully without any issues. The IOP for left and right eyes in the horse stable was 29.4 ± 8.8 mmHg and 29.6 ± 6.3 mmHg, respectively (*p* = 0.8). In the medical barn, the IOP for left and right eyes was 33.3 ± 6.7 mmHg and 35.1 ± 9.7 mmHg, respectively (*p* = 0.4). The tear production in the horse stable for left and right eyes was 22.3 ± 2.8 mm/min and 21.96 ± 4.8 mm/min, respectively (*p* = 0.76). In the medical barn, the tear production for left and right eyes was 24.3 ± 3.8 mm/min and 23.0 ± 4.0 mm/min, respectively (*p* = 0.1).

**FIGURE 1:**
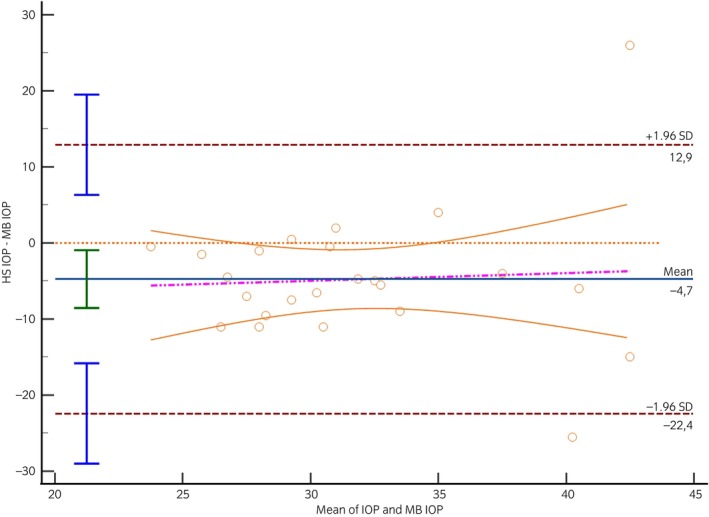
Bland–Altman plot. X‐axis: mean intraocular pressure (IOP) between horse stable (HS) and medical barn (MB), Y‐axis: difference between horse stable and medical barn, continuous blue line: mean difference between horse stable and medical barn, dashed brown lines: limits of agreement (= mean difference ± 1.96 standard deviations).

**FIGURE 2:**
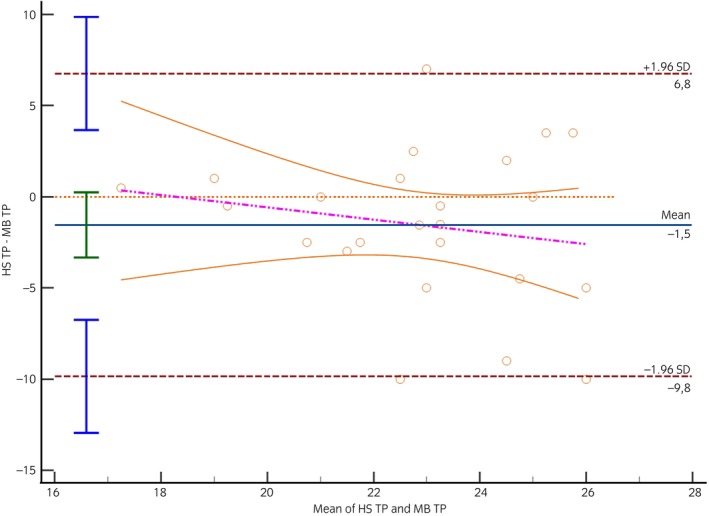
Bland–Altman plot. X‐axis: mean tear production (TP) between horse stable (HS) and medical barn (MB), Y‐axis: difference between horse stable and medical barn, continuous blue line: mean difference between horse stable and medical barn, dashed brown lines: limits of agreement (= mean difference ± 1.96 standard deviations).

As the IOP and tear production values did not differ between right and left eyes in the horse stable and the medical barn, the IOP and the tear production values of both eyes were used as a mean value for each horse. Notably, there was a significant increase in IOP in the medical barn (34.2 ± 6.8 mmHg) compared with the horse stable (29.5 ± 7.2 mmHg) (*p* = 0.02). However, no statistically significant difference in tear production was observed between horse stable (22.1 ± 2.8 mm/min) and medical barn (23.6 ± 3.4 mm/min) (*p* = 0.09). The standard error of the slope was 0.36 for the IOP measured in the medical barn, indicating a difference of −4.7 mmHg compared with the IOP measured in the horse stable (*p* = 0.02) (Figure 1). The fitted bias equation, *y* = −7.9350 + 0.1003*x* (Table 1), indicated that an increase in mean IOP corresponded to an increase in bias. The visual support from the Bland–Altman plot underscored the significance of considering potential biases and their impact on agreement when retrospectively comparing IOP measurements between the horse stable and medical barn. The standard error of the slope was 0.39 for the tear production measured in the medical, indicating a difference of −1.5 mm/min compared with the tear production measured in the horse stable (*p* = 0.09) (Figure 2). The bias was fitted to *y* = 6.1530 + −0.3367*x* (Table 2).

**TABLE 1:** Regression equations generated by the Bland–Altman regression analysis to compare bias of intraocular pressure (mmHg) measured from the horse stable (Y) and medical barn (X).

	Difference (mmHg)			
Mean (95% CI)	SEs	Lower limit (95% CI)	Upper limit (95% CI)	Regression equation
−4.74 (−8.54 to −0.93)	0.36	−22.4 (−29 to −15.81)	12.92 (6.33 to 19.52)	*y* = −7.9350 + 0.1003*x*, *p* = 0.02

Abbreviations: CI, confidence interval; SE, standard error of slope.

**TABLE 2:** Regression equations generated by the Bland–Altman regression analysis to compare bias of tear production (mm/min) measured from the horse stable (Y) and medical barn (X).

	Difference (mm/min)			
Mean (95% CI)	SEs	Lower limit (95% CI)	Upper limit (95% CI)	Regression equation
−1.54 (−3.33 to 0.24)	0.39	−9.85 (−12.9 to −6.75)	6.76 (3.66 to 9.86)	*y* = 6.1530 + −0.3367*x*, *p* = 0.09

Abbreviations: CI, confidence interval; SE, standard error of slope.

## 4 DISCUSSION

The main objective of our study was to compare IOP and tear production values measured in horses between a horse stable and a medical barn and it is the first investigation into IOP and tear production levels in horses across two distinct locations. The notable increase in IOP within the medical barn compared with the horse stable aligns with and accepts our hypothesis. The Bland–Altman analysis further highlighted the disparity in IOP measurements between the medical barn and the horse stable, indicating a difference of −4.7 mmHg in IOP, favouring the horse stable. Although the IOP values exhibited statistically significant differences, a variation of less than 5 mmHg is unlikely to be clinically relevant. Nevertheless, the findings suggest that the choice of environment can have a discernible impact on the physiological parameters of horses, specifically in relation to IOP. This is consistent with findings in other species, reinforcing the idea that stressors can influence IOP.^11^, ^12^ A study conducted in a veterinary hospital and farm reported the IOP of male horses as 30.0 ± 5.6 mmHg.^13^ In contrast, another study conducted in a horse stable documented IOP values for horses as 21.6 ± 2.45 mmHg.^14^ These previous observations correspond with our study's outcomes, providing support for our hypothesis that IOP measurements in the medical barn (34.2 ± 6.8 mmHg) are higher than those in the horse stable (29.5 ± 7.2 mmHg).

Previous studies have established the normal tear production values in horses as 22.9 ± 2.8 mm/min^15^ and 24.8 ± 4.8 mm/min.^16^ These values are consistent with our findings. In this study, tear production values exhibited no significant difference between the horse stable and the medical barn, challenging our initial hypothesis regarding tear production susceptibility to environmental influences. In contrast to IOP results, tear production did not show a notable distinction between locations, indicating a complex relationship between environmental stressors and ocular parameters. Furthermore, the Bland–Altman analysis indicates a tendency towards a −1.5 (mm/min) underestimation of tear production when measured in the horse stable, although this result did not reach statistical significance (*p* = 0.09). This suggests that while stress may impact IOP, its influence on tear production may differ, contributing to a more comprehensive understanding of ocular health in horses.

The Schirmer tear test‐1 is ideally conducted before any eye manipulation to reduce reflex tearing.^17^ However, it is essential to note that in our study, we employed rebound tonometry, which minimally interacts with the cornea. Additionally, we allowed a 5‐min interval between obtaining IOP values and conducting the STT measurements. This approach aimed to mitigate potential influences on the tear reflex, ensuring a more accurate assessment of tear production.

While the horses were deemed healthy based on the conducted examinations, it is crucial to acknowledge a significant limitation arising from the lack of a detailed posterior segment evaluation in the current study. The challenging conditions at the farm, coupled with logistical constraints, hindered a more comprehensive assessment of the horses' eyes. Another noteworthy limitation of this study is the absence of horses with ocular disorders. Ocular problems have the potential to either exaggerate or diminish the IOP values at levels that are clinically significant for ocular examinations. Another limitation of the current study is the absence of an assessment of the potential impact of transportation on IOP in horses. Prolonged transportation periods are recognised stressors for equines, leading to increased heart rate, mean arterial pressure, and cortisol levels.^18^, ^19^ These physiological changes induced by transportation‐related stress could influence ocular parameters. Horses often require transportation to veterinary clinics or hospitals, a factor that may introduce variations in IOP during clinical examinations, adding complexity to the interpretation of results. Future studies specifically addressing the effects of transportation‐related stress on ocular health parameters would enhance our understanding of factors influencing IOP in equine populations. While our study sheds light on the potential impact of transportation, future research endeavours should strive for a more comprehensive understanding by simultaneously investigating other examination‐related variables such as sedation and diurnal variation.

In conclusion, a statistically significant increase in IOP was noted in the medical barn compared with the horse stable, while tear production showed no significant variance between the two environments. The Bland–Altman analysis highlighted a discrepancy in IOP measurements in the horse stable, emphasising the potential influence of the environment on ocular parameters in horses.

## FUNDING INFORMATION

Not applicable.

## CONFLICT OF INTEREST STATEMENT

The authors declare no conflicts of interest.

## AUTHOR CONTRIBUTIONS

**Latif Emrah Yanmaz:** Writing – original draft; conceptualization; methodology; data curation; validation; investigation. **Harun Cinar:** Conceptualization; data curation; methodology; writing – review and editing; investigation; validation. **Nurefsan Buyukkaraca:** Conceptualization; investigation; validation; methodology; data curation. **Zehranur Kaya:** Conceptualization; investigation; data curation. **Mirkan Kosuncu:** Conceptualization; investigation; data curation.

## DATA INTEGRITY STATEMENT

Latif Emrah Yanmaz had full access to all the data in the study and takes responsibility for the integrity of the data and the accuracy of the data analysis.

## ETHICAL ANIMAL RESEARCH

The Local Board of Ethics Committee for Animal Experiments in Burdur Mehmet Akif Ersoy University approved to the study protocol (Decision No: 1043/2023).

## INFORMED CONSENT

Informed client consent was gained for each horse before inclusion into the study.

### PEER REVIEW

The peer review history for this article is available at https://www.webofscience.com/api/gateway/wos/peer-review/10.1111/evj.14067.

## Data Availability

The data that support the findings will be available in Google docs at https://docs.google.com/spreadsheets/d/1idZXIZJuSxtqtXX6pQQo6n3bWQVh8OeA/edit#gid=1719025261 following an embargo from the date of publication to allow for commercialization of research findings.
